# Artificial Intelligence for Perioperative Risk Prediction and Prevention in Cardiac Surgery: A Narrative Review and Proposed Conceptual Framework

**DOI:** 10.3390/jcm15145325

**Published:** 2026-07-08

**Authors:** Dimitrios E. Magouliotis, Serge Sicouri, Vasiliki Androutsopoulou, Alexandra Bekiaridou, Massimo Baudo, Thanos Athanasiou, Andrew Xanthopoulos, George C. Prendergast, Basel Ramlawi

**Affiliations:** 1 Department of Cardiac Surgery Research, Lankenau Institute for Medical Research, Wynnewood, PA 19096, USA; sicouris@mlhs.org (S.S.); massimo.baudo@icloud.com (M.B.); ramlawib@mlhs.org (B.R.); 2 Department of Cardiothoracic Surgery, Faculty of Medicine, University of Thessaly, Biopolis, 41110 Larissa, Greece; androutsopoulouvasiliki@uth.gr; 3 Northwell Health, New Hyde Park, New York, NY 11040, USA; ampekiaridou@gmail.com; 4 Elmezzi Graduate School of Molecular Medicine, Northwell Health, Manhasset, NY 11030, USA; 5 Department of Surgery and Cancer, Imperial College London, London SW7 2AZ, UK; t.athanasiou@imperial.ac.uk; 6 Department of Cardiology, Faculty of Medicine, University of Thessaly, Biopolis, 41110 Larissa, Greece; andrewvxanth@gmail.com; 7 Lankenau Institute for Medical Research, Wynnewood, PA 19096, USA; prendergast@limr.org; 8 Department of Cardiac Surgery, Lankenau Medical Center, Wynnewood, PA 19096, USA

**Keywords:** artificial intelligence, quality improvement, cardiac surgery, perioperative management, clinical outcomes

## Abstract

Cardiac surgery remains a high-risk, resource-intensive domain in which perioperative complications significantly influence clinical outcomes, institutional performance, and healthcare expenditure. Despite advances in technique and protocol standardization, contemporary perioperative management largely relies on static risk stratification and reactive quality assessment. This narrative review synthesizes the current evidence on artificial intelligence (AI) and machine learning for perioperative risk prediction in cardiac surgery, spanning acute kidney injury, mortality, prolonged mechanical ventilation, postoperative atrial fibrillation, and intensive care unit deterioration, and critically appraises the methodological limitations, validation gaps, and fairness concerns that constrain clinical translation. Across these applications, predictive models have demonstrated incremental discrimination over conventional risk scores, yet remain predominantly endpoint-specific, single-institution, and disconnected from prospective clinical implementation. Building on this evidence, we propose Preventive Cardiovascular Intelligence (PCInt) as one possible organizing framework that integrates predictive analytics, dynamic risk trajectory modeling, and structured quality improvement methodologies, and we outline how such a framework might be operationalized across the surgical lifecycle. PCInt is presented as a conceptual proposal requiring prospective validation rather than as a validated system. We conclude by discussing implementation barriers, regulatory and ethical considerations, and priorities for future research toward anticipatory, value-based perioperative cardiovascular care.

## 1. Introduction

Cardiac surgery remains one of the most complex and resource-intensive domains within contemporary cardiovascular care, serving as the definitive therapeutic modality for advanced valvular disease, coronary artery disease, aortic pathology, and heart failure. Despite remarkable progress in operative technique, myocardial protection, anesthetic management, and perioperative critical care, postoperative complications continue to exert a disproportionate influence on early and late clinical outcomes [1,2]. Acute kidney injury, prolonged mechanical ventilation, atrial fibrillation, bleeding requiring transfusion, low cardiac output syndrome, and sepsis represent interdependent adverse events that frequently propagate through sequential physiologic deterioration, therefore amplifying morbidity and mortality risk [3,4,5]. Importantly, these complications extend their impact beyond individual patients, contributing substantially to intensive care unit (ICU) utilization, prolonged hospital length of stay (LOS), readmission rates, and long-term healthcare expenditure [6,7]. In high-acuity service lines such as cardiac surgery, small variations in complication incidence translate into significant operational strain and financial variability, underscoring the dual clinical and institutional importance of perioperative risk control.

Risk stratification tools such as the Society of Thoracic Surgeons (STS) risk models and EuroSCORE II have improved perioperative prognostication and benchmarking by enabling structured comparison across institutions and facilitating informed consent discussions [8,9]. These models have provided an essential foundation for outcome transparency and quality measurement; however, these risk models remain predominantly static, estimating complication or mortality probability at a single preoperative time point. They do not account for dynamic intraoperative variables, evolving physiologic instability, or postoperative risk acceleration. Furthermore, traditional quality improvement paradigms in cardiac surgery, while increasingly sophisticated, often rely on retrospective data analysis, registry audits, and morbidity and mortality conferences that identify adverse events after their occurrence [10,11]. Although such mechanisms enhance accountability and promote system learning, they are inherently reactive and episodic, rather than continuously anticipatory. As perioperative data streams expand, including real-time hemodynamics, laboratory trends, perfusion metrics, imaging-derived phenotypes, and longitudinal registry outcomes, the gap between data availability and pro-active risk modulation becomes increasingly apparent.

In parallel, preventive cardiology has successfully reduced long-term cardiovascular morbidity through structured modification of risk factors such as dyslipidemia, hyper-tension, diabetes, and lifestyle behaviors [12,13]. This paradigm shift, extending from treating established disease to attenuating long-term risk trajectories, has transformed outpatient cardiovascular medicine. However, the conceptual architecture of prevention has not been systematically translated into the perioperative environment, where risk unfolds over compressed time horizons and complication cascades may evolve within hours. The rapid development of artificial intelligence (AI) and machine learning methodologies in cardiovascular medicine offers the potential to bridge this gap by enabling high-dimensional predictive modeling, early detection of physiologic deterioration, and integration of multimodal data streams [14,15,16]. In cardiac surgery, emerging AI applications have demonstrated improved prediction of mortality, acute kidney injury, and prolonged ventilation beyond conventional risk scores [17,18,19]. Yet, these efforts remain fragmented and predominantly endpoint-specific, lacking integration into a cohesive institutional framework that connects prediction with structured preventive intervention, governance structures, and resource optimization. Consequently, predictive analytics frequently exist in parallel to, rather than embedded within, perioperative management strategy.

To organize the evidence reviewed here and to illustrate how prediction might be linked to prevention, we introduce, in Section 4, a proposed conceptual framework termed Preventive Cardiovascular Intelligence (PCInt): an institutional perioperative model that integrates artificial intelligence, dynamic risk trajectory modeling, and structured quality improvement methodologies, intended to anticipate and modulate adverse events across the surgical lifecycle. We emphasize at the outset that PCInt is advanced as a conceptual synthesis and research agenda rather than as a validated or deployed system; no empirical evaluation of the integrated framework is presented, and the claims made for it are hypotheses requiring prospective testing. Unlike conventional risk assessment approaches that quantify probability at a single moment in time, PCInt conceptualizes perioperative risk as a dynamic and potentially modifiable process spanning preoperative optimization, intraoperative modulation, and postoperative stabilization. The remainder of this review surveys the existing evidence base before presenting this framework and the open questions it raises.

## 2. Materials and Methods

This article is a narrative review. We searched PubMed/MEDLINE, Embase, and the Cochrane Library for English-language publications from January 2010 to March 2026, supplemented by hand-searching of reference lists of included articles and relevant society guidelines. Search terms combined controlled vocabulary and free-text keywords across two thematic axes: (i) artificial intelligence, machine learning, deep learning, predictive modeling, neural networks, and early warning systems; and (ii) cardiac surgery, perioperative care, acute kidney injury, postoperative atrial fibrillation, mechanical ventilation, intensive care, mortality prediction, and risk stratification. Conventional risk models (Society of Thoracic Surgeons risk scores, EuroSCORE II) and learning health system concepts were searched as additional terms.

Studies were eligible if they addressed the development, validation, or clinical application of AI or machine learning methods for perioperative prediction or decision support in adult cardiac surgery, or provided conceptual or methodological context relevant to that aim. Priority was given to peer-reviewed original studies, systematic reviews, landmark methodological papers, and current clinical guidelines. We excluded non-English publications, conference abstracts without full text, and studies confined to non-cardiac surgical populations unless they provided foundational methodological or regulatory context. Because this is a narrative rather than systematic review, article selection involved author judgment regarding representativeness and relevance; we did not perform formal risk-of-bias scoring or quantitative synthesis, and the absence of a particular study should not be interpreted as a judgment on its quality. Given this design, the synthesis is intended to be illustrative of the state of the field rather than exhaustive.

## 3. Limitations of Current Risk Stratification and Reactive Perioperative Paradigms

To understand the value proposition of Preventive Cardiovascular Intelligence, it is necessary to map with precision the structural limitations of existing perioperative risk management, not as a critique of validated tools that have meaningfully advanced the field, but as a rigorous diagnostic of the architectural constraints that prevent their translation into preventive action.

### 3.1. Static Risk Models: Calibration at the Cost of Dynamism

The STS Predicted Risk of Mortality (PROM) score and EuroSCORE II represent the most widely deployed risk stratification instruments in cardiac surgery globally [2,3]. Both risk scores were developed from large national and international registry datasets, are regularly recalibrated, and provide clinically useful estimates of operative mortality for preoperative counseling, patient selection, and cross-institutional benchmarking [2,20,21,22,23,24]. Their validity for these purposes is not in question.

However, the fundamental design of both systems reflects an epistemological choice: the prediction of population-level outcomes from a preoperative snapshot of patient characteristics. Variables such as age, ejection fraction, prior cardiac surgery, renal function, and emergency status are captured at a single time point (the preoperative assessment) and generate a risk score that remains fixed thereafter [5]. This design creates three distinct clinical liabilities: inability to incorporate intraoperative events, failure to account for dynamic postoperative physiologic evolution, and absence of actionable clinical triggers. Static models categorize risk; however, they do not direct preventive responses [4,14,25].

### 3.2. Fragmented Data Streams: The Architecture of Disconnection

Modern cardiac surgical ICUs generate an extraordinary volume of physiologic data. Continuous hemodynamic monitoring (including arterial lines, central venous pressure, near-infrared spectroscopy) produces high-frequency data streams recorded but rarely analyzed in integrated fashion [26]. Laboratory trajectories (e.g., serum creatinine, lactate, hemoglobin, coagulation parameters, brain natriuretic peptide) evolve continuously and contain predictive information that point-in-time review cannot fully exploit [27]. Cardiopulmonary bypass perfusion data (e.g., flows, pressures, temperatures, oxygen delivery indices) represent an entirely distinct physiologic domain with outcome implications that remain poorly operationalized in quality systems [28].

The challenge is not data scarcity but rather data integration. Hemodynamic signals reside in bedside monitors or proprietary vendor platforms. Laboratory values are housed in electronic health record (EHR) systems with variable structured data quality [29]. The result is often a perioperative informational ecosystem characterized by abundant data and fragmented insight. For instance, clinicians review individual signals sequentially, applying experiential heuristics in the absence of integrated analytical infrastructure [30].

### 3.3. Reactive Quality Improvement: The Retrospective Trap

Quality improvement (QI) in cardiac surgery has historically been conducted through retrospective mechanisms: morbidity and mortality (M&M) conferences reviewing adverse outcomes after their occurrence, database submissions to the STS National Database for annual benchmarking, and root cause analyses triggered by sentinel events [13,31]. These mechanisms have produced meaningful improvements in outcomes over the past few decades. The steady reduction in CABG mortality over the past 30 years remains the most characteristic example and attests to the value of systematic outcome monitoring [32]. Nonetheless, retrospective QI is structurally limited to learning from complications that have already occurred, rather than preventing those that are predictably imminent.

This distinction reflects a fundamental asymmetry between the informational richness of modern perioperative data and the analytical infrastructure deployed to interpret it. Bridging this gap requires more than just better data collection; it requires a conceptual reorientation of what perioperative quality improvement is and what it can achieve. Learning health system frameworks offer the most promising conceptual model for this reorientation [33,34]. The structural differences between traditional static risk stratification models and the proposed AI-driven PCInt framework across nine operational domains (including timing, data inputs, actionability, feedback loops, and clinical culture) are juxtaposed in Table 1.

## 4. Artificial Intelligence in Cardiovascular and Surgical Medicine

### 4.1. Overview of AI Methodologies in Cardiovascular Medicine

Artificial intelligence, broadly defined as the application of computational algorithms to perform tasks that would otherwise require human cognitive capability, encompasses a spectrum of methodological approaches increasingly relevant to cardiovascular medicine [6]. Supervised learning algorithms, including logistic regression with regularization, gradient boosting machines (XGBoost, LightGBM), and random forests, have in several reported series achieved improved discrimination relative to traditional regression-based risk scores across multiple cardiovascular outcomes, although the magnitude of any advantage varies with dataset, outcome, and validation design [8,15]. These models excel at capturing nonlinear relationships, interaction effects, and high-dimensional variable structures poorly handled by conventional regression approaches [9].

Deep learning represents a hierarchically structured neural network-based approach capable of autonomous feature extraction from high-dimensional inputs. It has extended AI capabilities into domains previously inaccessible to conventional machine learning (ML) [7]. Convolutional neural networks (CNNs) applied to electrocardiographic and echocardiographic data have enabled automated arrhythmia detection, left ventricular function assessment, and valvular disease classification with performance approaching or exceeding expert interpretation [19]. Recurrent neural networks (RNNs) and long short-term memory (LSTM) architectures, designed for sequential data streams, are particularly suited to the high-frequency continuous physiologic monitoring data generated in cardiac surgical ICUs [21].

Natural language processing (NLP), a domain of AI focused on the interpretation of unstructured human language, has enabled extraction of clinically meaningful information from free-text operative notes, discharge summaries, and clinical documentation [35]. Predictive analytics, the integrative application of ML methodologies to forward-looking clinical prediction, represents the most direct translational interface between AI capability and perioperative outcome improvement [29,36].

### 4.2. AI Applications in Cardiac Surgery: Current Evidence

The application of AI to cardiac surgical outcomes prediction has produced a substantial and accelerating body of literature [4]. Acute kidney injury (AKI), affecting 20–40% of cardiac surgery patients and independently associated with increased mortality, prolonged ICU stay, and long-term renal dysfunction, has been the most extensively studied prediction target [27]. Multiple studies have reported Area Under the Receiver Operating Characteristic Curve (AUROC) values of 0.80–0.90 for ML-based AKI prediction models incorporating preoperative, intraoperative, and early postoperative variables, in most series exceeding the discrimination of traditional risk scores; these figures reflect predictive discrimination in development or internal-validation cohorts rather than demonstrated improvement in clinical outcomes, and external validation remains limited [37,38,39].

Mortality prediction models leveraging ML have similarly demonstrated incremental performance over STS PROM, particularly in high-complexity and emergent operative scenarios where traditional models are less well-calibrated [16,17,18]. Prolonged mechanical ventilation in cardiac surgery represents a major driver of ICU resource consumption and ventilator-associated complications, and has been successfully predicted with ML models achieving sensitivity exceeding 80% at clinically actionable thresholds [40].

Deep learning applied to continuous ECG surveillance has demonstrated capacity for real-time prediction of postoperative atrial fibrillation (POAF), affecting 20–50% of patients after cardiac surgery [41,42], up to 24 h before clinical onset [19,20,43]. ICU deterioration detection, leveraging multiparametric physiologic data streams through LSTM-based architectures, has achieved early warning performance superior to conventional Modified Early Warning Score (MEWS) and National Early Warning Score (NEWS) systems in cardiac surgical populations [26]. The breadth of AI applications in cardiac surgery, spanning AKI prediction, mortality modeling, prolonged ventilation, POAF surveillance, ICU deterioration detection, and perfusion optimization, together with their respective methodologies and reported performance metrics, is summarized in Table 2 [16,17,18,19,20,26,29,35,36,40,41,42,43,44,45,46,47,48,49,50,51,52,53,54,55,56].

### 4.3. Emerging Data Ecosystems: The Infrastructure of Preventive Intelligence

The translational potential of AI in cardiac surgery is inseparable from the maturation of the data ecosystems that supply it. EHRs, while highly variable in structured data quality, are increasingly implementing standardized clinical data architectures (HL7 FHIR, OMOP Common Data Model) that facilitate multisite data aggregation and model development [29,30]. Real-time physiologic monitoring platforms capable of streaming waveform-level data at 125–500 Hz are increasingly interfaced with hospital middleware systems, creating the technical substrate for continuous AI-mediated surveillance [26].

Wearable and implantable sensor technologies represent a rapidly advancing frontier for preoperative risk trajectory characterization and postdischarge surveillance [44]. The proliferation of perioperative big data repositories, including multi-institutional cardiac surgical databases with longitudinal linkage to administrative, laboratory, and imaging data, creates unprecedented opportunities for federated learning approaches that preserve patient privacy while enabling cross-institutional analytical collaboration [30]. Despite this expanding infrastructure, a critical translational gap persists: AI tools in cardiac surgery remain primarily developed as standalone research artifacts, disconnected from prospective clinical implementation, institutional quality frameworks, and the decision-support workflows through which perioperative care is delivered [4,22].

## 5. A Proposed Conceptual Framework: Preventive Cardiovascular Intelligence (PCInt)

### 5.1. Foundational Definition and Philosophical Basis

A note on terminology is warranted before the framework is described. Because PCInt has not been built, deployed, or evaluated, the following two sections use conditional and future-oriented language (for example, would compute, would generate, would activate, would require validation) to describe its intended design properties. Every behavior attributed to PCInt below should be read as a proposed capability of a hypothetical system that would require prospective development and validation, not as an observed capability of an existing one. Where the present tense is unavoidable for readability, it denotes a design intention rather than a demonstrated function. The empirical status of the framework, and the evidence that would be required to substantiate these design intentions, is addressed in Section 8 and Section 9.

Preventive Cardiovascular Intelligence (PCInt) is defined as an AI-driven institutional framework integrating predictive analytics, continuous perioperative data streams, and structured quality improvement strategies to proactively stabilize patient risk trajectories across the surgical encounter. This definition is deliberate in every element. It is AI-driven because the predictive substrate demands machine learning’s capacity to model high-dimensional, nonlinear, time-varying physiologic complexity. It is institutional because the framework’s efficacy depends on organizational embedding rather than individual clinical heroics. It is continuous perioperative because the unit of observation is the entire surgical encounter, from preoperative risk phenotyping through postdischarge surveillance, rather than a single clinical snapshot. It is also proactive stabilization because the intended output is risk reduction, initiated before complications clinically manifest, rather than risk documentation.

The philosophical basis of PCInt draws on a foundational distinction in clinical epistemology: the difference between prognosis and prevention. Risk stratification in its traditional form is a prognostic exercise that estimates the probability of an adverse outcome given a patient’s baseline characteristics, but it does not alter the probability it estimates [14]. PCInt is intended to extend this prognostic stance rather than to displace it. Validated static scores such as the STS models and EuroSCORE II remain essential for operative-risk estimation, preoperative counseling, benchmarking, and quality assessment, and PCInt is proposed as a complement to these tools, not a replacement for them. The premise of the framework is that the clinical value of a risk estimate is realized most fully when the estimate is linked to a clinical response: a probability that prompts a preventive action may avert an outcome that the same probability, used only for documentation and benchmarking, would merely record. PCInt therefore reframes prediction as the first step in a clinical process designed to prevent the predicted outcome: identifying the patient at risk for AKI, stroke, or respiratory failure, and intervening early enough to alter the trajectory before the complication occurs [6,22].

This reframing is not merely conceptual. It has structural consequences for every component of the framework: the temporal architecture of the predictive models (continuous, not single-point); the design of the alert system (trajectory-sensitive, not threshold-only); the structure of the decision support protocols (action-linked, not advisory only); and the governance of the institutional quality system (prospective, not retrospective). PCInt is therefore not a product that can be purchased and deployed; it is a clinical philosophy that must be built, embedded, and continuously maintained. In this context, it is this architectural depth, rather than any single predictive model, that is intended to distinguish the proposed framework from existing standalone AI applications in cardiac surgery [33,34].

### 5.2. Theoretical Antecedents and Conceptual Positioning

PCInt does not emerge from a conceptual vacuum. It synthesizes and extends three established frameworks in clinical medicine and health systems science. The first one is the learning health system paradigm, the model in which routine clinical care continuously generates the data infrastructure for system improvement, and in which the gap between evidence generation and clinical practice is progressively narrowed through embedded feedback mechanisms [33,34]. PCInt operationalizes this paradigm specifically for the high-acuity, data-dense environment of cardiac surgical care, where the combination of procedural volume, outcome stakes, and monitoring infrastructure creates ideal conditions for a self-improving clinical system.

The second framework is precision medicine, representing the principle that clinical decisions should be individualized to the biological, physiological, and contextual characteristics of the specific patient, rather than derived from population averages [7]. PCInt applies precision medicine principles to the perioperative domain: replacing population-level risk categorization with patient-specific, moment-by-moment risk quantification, therefore replacing protocol-based management with individually triggered preventive interventions calibrated to the patient’s actual physiologic trajectory.

The third framework is predictive analytics in critical care. This body of evidence demonstrates that early warning systems using multiparametric physiologic data can identify clinical deterioration significantly earlier than conventional clinical recognition. This enables earlier intervention and improved outcomes [26]. PCInt extends this critical care concept into the entire perioperative surgical process, integrating preoperative phenotyping, intraoperative monitoring, and postoperative surveillance into a unified, temporally continuous predictive architecture that no existing critical care early warning system encompasses.

### 5.3. The PCInt Data Architecture: Multimodal Perioperative Integration

The operational substrate of PCInt is a unified, real-time, multimodal data architecture that harmonizes the distinct informational ecosystems of the cardiac surgical encounter into a single analytical environment. This architecture spans five data domains, each contributing non-redundant predictive signal that current fragmented systems cannot jointly exploit [29,30].

The first domain is the structured clinical record including preoperative demographics, comorbidities, functional status, medication lists, laboratory values, and imaging findings. Although the domain is already captured by STS and EuroSCORE, it could be exploited by PCInt with far greater variable dimensionality and ML-based feature interaction modeling [2,3,17].

The second domain is the intraoperative perfusion and anesthesia record including cardiopulmonary bypass time and temperature management, cross-clamp duration, flow index trajectories, vasopressor and inotrope administration, blood product utilization, and hemodynamic event logs from the anesthesia information management system. This is a domain of great predictive value that is not incorporated into any existing risk score [28,45].

The third domain is continuous physiologic monitoring including high-frequency waveform data from arterial lines, central venous and pulmonary artery catheters, near-infrared spectroscopy cerebral oximetry, pulse oximetry, and capnography. These streams generate data at 125–500 Hz and contain temporal patterns of hemodynamic instability, respiratory compromise, and end-organ perfusion inadequacy. These patterns can be detected by ML algorithms significantly before conventional clinical alert thresholds are crossed [26,44].

The fourth domain is laboratory and biomarker trajectories including serial serum creatinine, lactate, hemoglobin, troponin, brain natriuretic peptide, coagulation parameters, and acid-base indices whose rate of change, rather than absolute values, carries the strongest predictive signal for major postoperative complications [27,39].

**Figure 1:**
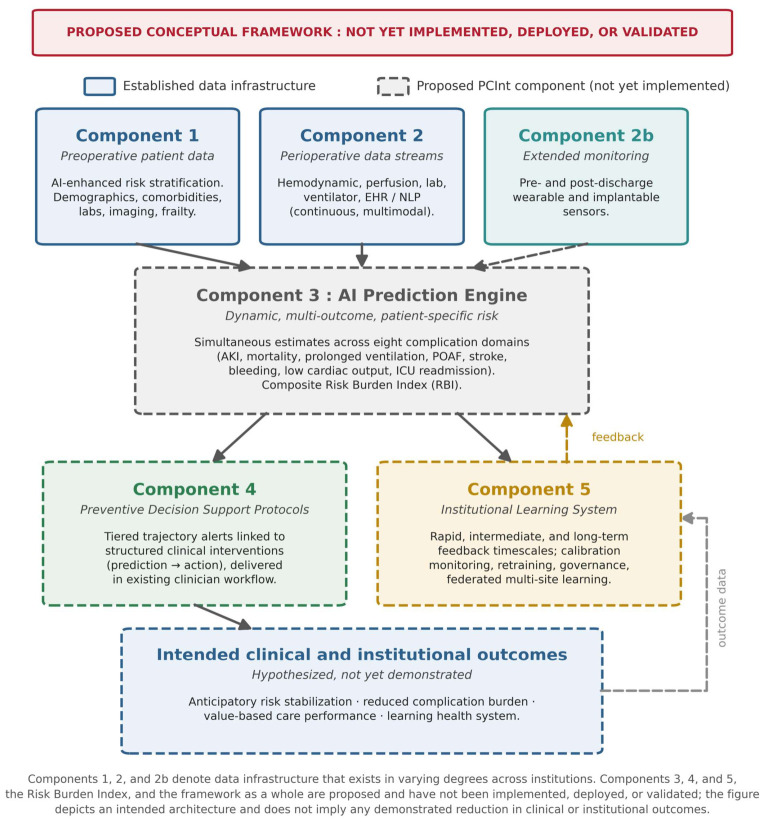
The Preventive Cardiovascular Intelligence (PCInt) Framework. Five integrated components are depicted: preoperative patient data input and AI-enhanced risk stratification (Component 1); continuous perioperative multimodal data streams encompassing hemodynamic, perfusion, laboratory, ventilator, and EHR/NLP domains (Component 2); extended pre- and post-discharge monitoring via wearable and implantable sensors (Component 2b); the central AI Prediction Engine generating simultaneous, patient-specific risk estimates across eight complication domains with a composite Risk Burden Index (Component 3); Preventive Decision Support Protocols linking tiered trajectory alerts to structured clinical interventions (Component 4); and the Institutional Learning System operating across rapid, intermediate, and long-term feedback timescales (Component 5). Arrows indicate directional data flow, decision support activation, and learning feedback pathways. Components 1, 2, and 2b correspond to data-infrastructure elements that already exist in varying degrees across institutions, whereas Components 3, 4, and 5, together with the Risk Burden Index and the integrated framework as a whole, are proposed and have not been built, deployed, or validated. The figure depicts an intended architecture; it does not represent an implemented system and does not imply any demonstrated reduction in clinical or institutional outcomes. AKI, Acute Kidney Injury; AUROC, Area Under the Receiver Operating Characteristic Curve; EHR, Electronic Health Record; ICU, Intensive Care Unit; LOS, Length of Stay; LSTM, Long Short-Term Memory; MAP, Mean Arterial Pressure; NIRS, Near-Infrared Spectroscopy; NLP, Natural Language Processing; PDSP, Preventive Decision Support Protocol; POAF, Postoperative Atrial Fibrillation; RBI, Risk Burden Index; SHAP, SHapley Additive exPlanations; STS, Society of Thoracic Surgeons; TCN, Temporal Convolutional Network; XAI, Explainable Artificial Intelligence; XGBoost, Extreme Gradient Boosting.

The fifth domain is unstructured clinical documentation including nursing assessments, physician notes, respiratory therapist records, and pharmacist interventions. All these documents are processed through NLP to extract clinically actionable signals (pain descriptors, neurological assessments, fluid intake language, mobility notations) that are absent from structured data fields [35]. The integration of these five domains through a unified data pipeline (harmonized to a common temporal index, quality-flagged for missingness and physiologic plausibility, and fed continuously to the PCInt prediction engine) constitutes the most technically demanding component of the entire framework [29,46]. The integrated architecture of PCInt, encompassing its five data domains, AI prediction engine, preventive decision support protocols, and institutional learning system, is depicted schematically in Figure 1.

### 5.4. Predictive Risk Modeling: Dynamic, Multi-Outcome Risk Quantification

The core analytical layer of PCInt is a continuously updated, multi-output predictive risk engine. Unlike single-outcome ML models, which predict one complication in isolation and are therefore clinically incomplete, the PCInt risk engine generates simultaneous, patient-specific probability estimates across a defined panel of major outcome domains These include AKI, in-hospital mortality, prolonged mechanical ventilation (beyond 24 h), stroke or neurological injury, POAF, bleeding requiring reoperation, low cardiac output syndrome, and unplanned ICU readmission [16,17,18,27].

This multi-outcome architecture is clinically relevant because major complications in cardiac surgery are generally not independent events. Instead, they are physiologically interconnected, often sharing upstream causal pathways, such as hemodynamic instability, systemic inflammatory response, and renal hypoperfusion, which are more predictable in aggregate than individually. A patient exhibiting concurrent early signals of AKI, low cardiac output, and prolonged ventilation risk is not three patients with three isolated problems. The co-occurrence pattern itself carries diagnostic and prognostic information that single-outcome models cannot capture [39,47].

Model architectures within the PCInt risk engine would be selected by data type and temporal structure. Gradient boosting ensembles (XGBoost, LightGBM) would be appropriate for structured tabular data at defined assessment time points, offering high discriminative performance, robust handling of missing data, and native feature importance outputs that support explainability requirements [8,37]. Long short-term memory (LSTM) and temporal convolutional network (TCN) architectures would be suited to sequential high-frequency physiologic streams, capturing deterioration patterns in hemodynamic and respiratory data with a temporal resolution that tabular models cannot achieve [21]. Transformer-based architectures, increasingly validated in clinical NLP and multimodal EHR analysis, would be candidates for documentation integration and for modeling long-range temporal dependencies across the full perioperative encounter [7,35]. Critically, each model would be evaluated not only for discrimination (AUROC and area under the precision-recall curve) but also, and with equal weight, for calibration. Calibration assessment should not rely primarily on the Hosmer-Lemeshow statistic, which has well-recognized limitations including sensitivity to sample size and arbitrary grouping; preference should instead be given to calibration-in-the-large, calibration slope, calibration plots, the Brier score, and expected calibration error, complemented by decision-analytic measures such as net benefit on decision-curve analysis where a clinical action threshold can be defined. This emphasis matters because a model that discriminates well but is systematically miscalibrated would generate misleading probability outputs that undermine clinical decision-making in precisely the scenarios where accuracy matters most [14,48].

### 5.5. Risk Trajectory Analysis: From Probability to Velocity

The distinction between a risk probability and a risk trajectory is not semantic. In fact, it is clinically fundamental. A probability is a point estimate that answers the question: “What is this patient’s current likelihood of complication X?” A trajectory is a dynamic signal that answers the questions: “How is the likelihood changing, and how quickly?” PCInt’s risk trajectory analytic layer introduces a third dimension, that of the temporal velocity of risk change, absent from every existing perioperative risk management system [26].

Formally, the PCInt trajectory layer would compute, at each monitoring interval, both the current risk probability estimate and its first derivative over a configurable time window (typically 1–6 h in the ICU setting). Three trajectory classes would be defined. A stable trajectory (probability within ±5% of the preceding interval estimate) would generate no alert and would require no protocol activation. An escalating trajectory (probability rising at a rate exceeding a predefined velocity threshold) would generate a tiered alert proportional to both current risk level and rate of escalation, activating the corresponding preventive decision support protocol. A de-escalating trajectory, or probability falling after an intervention, would generate a protocol stand-down signal and would contribute a positive training example to the institutional learning system, attributing outcome improvement to the triggered intervention [26,33].

**Figure 2:**
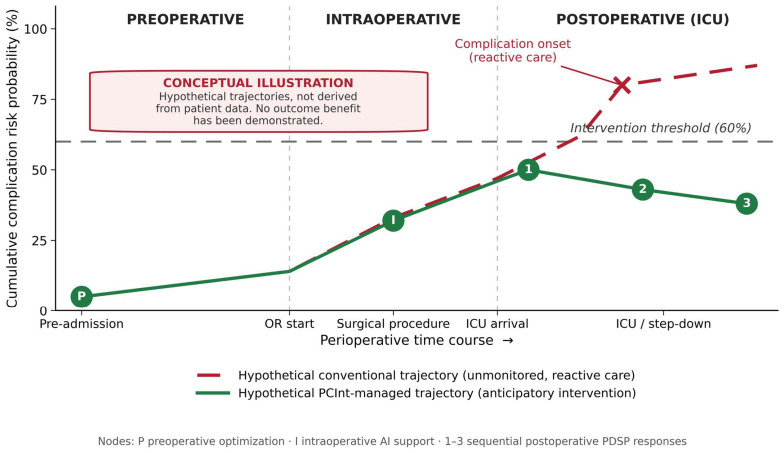
Perioperative Risk Trajectory Model under the PCInt Framework. This figure is a conceptual schematic intended to illustrate the framework’s logic; the curves are illustrative and are not derived from real patient data or from any empirical analysis. Cumulative complication risk probability (%) is plotted across three perioperative phases: preoperative (pre-admission through OR start), intraoperative (surgical procedure), and postoperative (ICU/step-down). The dashed red line represents a hypothetical unmonitored conventional trajectory, which crosses the intervention threshold (60% cumulative risk probability) and culminates in complication onset under reactive care. The solid green line represents a hypothetical PCInt-managed trajectory, in which sequential alert activations, namely preoperative optimization (Node P), intraoperative AI support (Node I), and three postoperative PDSP responses (Nodes 1–3), attenuate risk escalation and maintain the trajectory below the intervention threshold. The key concept panel emphasizes that PCInt would monitor the velocity of risk change rather than its absolute level, to enable anticipatory intervention across three trajectory classes: Stable, Escalating, and De-escalating. Both trajectories are illustrative and are not derived from patient data; the figure depicts the intended logic of the framework and does not represent measured outcomes or any demonstrated comparative benefit. AKI, Acute Kidney Injury; DO_2_, Oxygen Delivery Index; ICU, Intensive Care Unit; MAP, Mean Arterial Pressure; OR, Operating Room; PDSP, Preventive Decision Support Protocol; POAF, Postoperative Atrial Fibrillation; RAAS, Renin–Angiotensin–Aldosterone System.

This trajectory classification system is intended to have two properties that, if validated, would differentiate it from threshold-based early warning systems. First, it is anticipatory: rather than waiting for a static threshold to be crossed, it detects the velocity of deterioration, identifying patients heading toward a dangerous state before they arrive there, and preserving the intervention window. Second, it is adaptive: trajectory thresholds can be calibrated to individual patient baseline risk profiles. For example, a rising trajectory in a patient with pre-existing chronic kidney disease generates a different alert sensitivity than the same velocity change in a patient with normal baseline renal function [27,47]. Figure 2 illustrates the temporal evolution of cumulative complication risk probability under conventional unmonitored care compared to PCInt-managed trajectories, which includes preoperative optimization, intraoperative AI support, and sequential postoperative alert activations.

### 5.6. Preventive Decision Support: Closing the Prediction-to-Action Gap

The most consequential, and consistently absent component in existing AI applications in cardiac surgery is the link between prediction and action. Published ML models in cardiac surgery invariably conclude with a discrimination statistic and a comparison to conventional risk scores. Nonetheless, they do not specify how clinicians should use the generated probability estimate, when they should use it, or how doing so will alter the risk trajectory. This omission is not accidental. It reflects a category error in which the research community has treated prediction as an endpoint rather than as a means to the actual goal of prevention [22,23,49].

PCInt operationalizes the prediction-to-action link through a structured library of Preventive Decision Support Protocols (PDSPs): predefined, evidence-based clinical intervention pathways, each linked to a specific outcome domain, triggered at a specific trajectory alert tier, and structured to deliver actionable clinical options, not merely informational alerts, to the responsible clinician within the existing workflow. PDSPs are intentionally designed as clinical prompts, not algorithmic mandates: they do not override clinician judgment but redirect clinical attention, present option sets, and document the clinician’s response for outcome attribution analysis [22,29].

The following examples are illustrative and institution-specific rather than prescriptive, and any alert-triggered intervention bundle would require multidisciplinary co-design, local adaptation, prospective evaluation, and safety monitoring before clinical use. For the AKI domain, a Tier 1 escalating trajectory alert might activate an AKI Prevention Protocol: automatic medication reconciliation review for nephrotoxin exposure (NSAIDs, aminoglycosides, contrast agents, RAAS inhibitors), hemodynamic optimization target adjustment (for example, a higher MAP target in selected at-risk patients), and automated urine output monitoring frequency escalation. A Tier 2 alert might additionally prompt nephrology consultation notification and activation of a renal protection bundle (fluid resuscitation optimization, diuretic dose adjustment, avoidance of further nephrotoxin exposure). For the prolonged ventilation domain, an escalating trajectory alert might prompt spontaneous awakening trial scheduling, sedation lightening protocol initiation, and physiotherapy consultation for early mobilization planning [27,40]. For the POAF domain, early trajectory escalation might prompt consideration of a prophylactic amiodarone protocol, electrolyte repletion verification, and intensivist notification for rhythm monitoring augmentation [41,42]. The specific thresholds, agents, and targets cited here are intended as worked examples; they are not treatment recommendations and would themselves require validation.

PDSPs are maintained as living clinical documents, updated at each institutional learning cycle to incorporate new evidence, outcome attribution data from preceding deployment periods, and institutional clinical culture adaptations identified through structured clinician feedback. This version-controlled protocol library would accumulate clinical evidence and institutional refinement over time, with specificity increasing as more cases are processed [33].

### 5.7. The Human-AI Interface: Workflow Design and Clinician Integration

The clinical impact of any AI-driven decision support system is ultimately determined not by its predictive accuracy but rather by the quality of its integration into clinical workflow, the degree to which it provides actionable intelligence at the right moment to the right clinician in the right format, without imposing cognitive burden that exceeds its informational value. Alarm fatigue, the desensitization of clinical teams to monitoring alerts through excessive, low-specificity notification, is among the most documented and consequential failures of existing ICU surveillance technology, and represents the primary clinical risk of poorly designed PCInt deployment [26,51].

PCInt’s human-AI interface is architected around four workflow integration principles. First, contextual embedding: PCInt outputs are delivered within the clinician’s existing workflow environment rather than requiring navigation to a separate platform. Second, tiered alert design: only Tier 2 and Tier 3 trajectory alerts generate active notification (push alerts); Tier 1 signals are surfaced passively in the clinical dashboard, visible on demand but not interrupting. This tiering substantially reduces alert burden while preserving sensitivity for clinically significant deterioration [22,29].

Third, explainability at the point of care: each PCInt alert is accompanied by a patient-specific SHAP-derived feature attribution display, a ranked list of the physiologic and clinical variables most responsible for the current risk estimate, rendered as a visual contribution chart that communicates prediction rationale in clinical language rather than mathematical notation. This explainability interface directly addresses the most consistently cited barrier to AI adoption in critical care, that is, the clinician’s inability to understand or trust the basis for an algorithmic recommendation [51,52]. Fourth, structured response capture: when a clinician receives a PCInt alert, the interface presents a brief response menu (intervention initiated, intervention deferred with reason, alert dismissed with reason) whose completion is required to close the alert. This response capture serves dual purposes: it ensures documentation of clinical decision-making for medico-legal purposes, and it generates the labeled outcome data required for the institutional learning system’s model retraining and protocol effectiveness evaluation [33].

### 5.8. Multi-Outcome Prioritization: The Risk Burden Index

In the context of a multi-outcome PCInt risk engine generating simultaneous probability estimates across eight or more complication domains, a clinical prioritization mechanism would be required to prevent alert proliferation and to direct clinical attention toward the most consequential concurrent risks. As one candidate mechanism, PCInt proposes a Risk Burden Index (RBI): a composite metric that would integrate the current probability estimates across all active outcome domains, weighted by the clinical severity and healthcare utilization cost of each outcome, to generate a single patient-level risk acuity score intended to support cross-patient prioritization at the unit or program level [12,27]. The RBI is advanced here as a proposed research construct, not as a validated or simulated metric; the formulation below is intended to make the proposal concrete and testable rather than to specify a finished instrument.

The RBI would be computed as a weighted sum: RBI = Σ(p_i_ × w_i_), where p_i_ is the current probability estimate for outcome domain i and w_i_ is the clinical severity weight for that domain, derived from a combination of attributable mortality, attributable LOS extension, and attributable cost from the institutional and published literature. Mortality carries the highest severity weight, followed by stroke, AKI requiring renal replacement therapy, low cardiac output syndrome, prolonged mechanical ventilation, POAF, bleeding requiring reoperation, and unplanned ICU readmission; this ordering is offered as one candidate weighting scheme rather than a definitive one [2,12,41].

Several methodological questions would have to be resolved before the RBI could be regarded as a usable metric, and we flag them explicitly rather than presupposing their solution. First, the derivation and validation of the severity weights are non-trivial: weights drawn from attributable mortality, length-of-stay extension, and cost could be estimated from registry and institutional data, but the appropriate data source, the relative weighting of mortality against morbidity, length of stay, cost, and patient-centered outcomes, and the method of validating the resulting scale all remain open and value-laden choices. Second, the weights would in all likelihood be institution-specific, because attributable cost and resource use vary with case mix, payer environment, and local practice; a weighting scheme calibrated at one center may not transfer to another, and whether weights should be local, pooled, or hybrid is itself an empirical question. Third, the component probabilities are not independent. Because major cardiac surgical complications share upstream physiology and frequently co-occur, a simple weighted sum risks double-counting correlated risk and overstating aggregate burden; a defensible index would need to account for the covariance structure among outcomes (for example, through correlation-aware aggregation or a jointly modeled multi-outcome formulation) rather than treating domains as additive. Fourth, the index carries fairness implications: if an RBI were used to allocate ICU attention or institutional resources, any differential miscalibration across demographic or socioeconomic subgroups could translate directly into inequitable allocation, so subgroup calibration and equity auditing would be prerequisites rather than refinements. For all of these reasons, the RBI should be read as a hypothesis-generating construct whose feasibility, weighting, and behavior would require explicit simulation against existing datasets and prospective evaluation before any clinical use.

The RBI would, if validated, support two applications that single-outcome models cannot. First, real-time unit-level acuity ranking: displayed on the ICU charge nurse and attending intensivist dashboard, the RBI could allow identification of the highest-risk patients at any moment, supporting rational allocation of intensivist rounding time, nursing attention, and rapid response resources across a multi-patient unit. Second, programmatic outcome monitoring: aggregate RBI trends across the full surgical census, tracked over rolling time windows, could provide an early signal of unit-level outcome deterioration, potentially identifying adverse institutional trends earlier than conventional retrospective outcome reporting [13,31,33].

### 5.9. Institutional Learning Systems: The Self-Improving Clinical Engine

The fourth and architecturally integrative component of PCInt is the institutional learning system, the mechanism by which every clinical encounter processed through the framework generates information that improves the framework’s future performance. Without this component, PCInt would be a sophisticated but static clinical tool: accurate within the bounds of its training data, but progressively miscalibrated as patient populations, clinical practices, and institutional processes evolve over time. The learning system is what transforms PCInt from a deployed AI product into living clinical intelligence that increases in value with each patient it monitors [33,34].

The institutional learning system operates across three feedback timescales. At the rapid feedback timescale (daily to weekly), automated model monitoring pipelines track discrimination and calibration statistics on incoming prediction-outcome pairs, flagging performance degradation that meets predefined drift thresholds and triggering automated model retraining using updated training datasets that incorporate recent institutional cases. At the intermediate timescale (monthly), structured outcome attribution analyses evaluate the association between PDSP protocol activations and subsequent outcome improvements in treated versus untreated (alert-deferred) patient cohorts, generating evidence for protocol effectiveness, which is then fed back into the PDSP library maintenance process [34,53].

At the long feedback timescale (quarterly to annual), PCInt performance metrics (model AUROC, calibration error, alert utilization rates, protocol adherence rates, and risk-adjusted outcome improvement attributable to PCInt deployment) are reviewed at structured quality governance meetings involving cardiac surgery, cardiac anesthesia, critical care, informatics, and quality leadership. This governance cadence ensures that PCInt remains a clinically accountable system, that is one whose performance is regularly audited, whose failures are systematically identified, and whose improvements are systematically implemented, consistent with the highest standards of clinical quality management and AI/ML regulatory compliance [33,54].

In federated multi-institutional deployments, the learning system extends beyond the single institution to incorporate cross-site model performance data, enabling global model updates that reflect the aggregate experience of all participating programs while preserving individual site data sovereignty through federated learning protocols [46]. This federated learning architecture is clinically significant: rare outcome events such as stroke, reoperation for bleeding, and in-hospital mortality occur at insufficient frequency at individual institutions to provide the statistical power required for stable model calibration in high-risk subgroups. Multi-site federated learning could in principle resolve this statistical limitation by enabling larger, more representative training data while preserving site data sovereignty [30,46].

## 6. A Proposed Perioperative Implementation Model in Cardiac Surgery

### 6.1. Preoperative Phase: AI-Enhanced Risk Stratification and Optimization

In the preoperative phase, PCInt extends conventional risk stratification by incorporating ML-based risk modeling as a complement to STS PROM and EuroSCORE II [2,3,4]. AI-enhanced preoperative risk assessment integrates a broader variable set than conventional scoring allows, including frailty indices, functional capacity metrics, detailed comorbidity trajectories, body composition parameters from available imaging, and pharmacological risk modifiers [17,55]. The output is not a single summary score but a multidimensional risk profile that identifies individual high-probability outcome domains and generates a prioritized optimization agenda.

Crucially, the preoperative phase of PCInt is prescriptive, not just predictive. AI-identified modifiable risk factors trigger structured prehabilitation protocols including anemia optimization, renal protection strategies, nutritional optimization, smoking cessation, and cardiac rehabilitation. The concept of “risk stabilization”, which reduces preoperative risk trajectory prior to the surgical exposure, is central to the PCInt philosophy and distinguishes it from risk scoring systems that accept baseline risk as fixed [14,47].

### 6.2. Intraoperative Phase: Real-Time Risk Modulation

The intraoperative phase of PCInt leverages the integration of anesthesia information management systems (AIMS), perfusion documentation platforms, and real-time physiologic monitoring to enable continuous risk state estimation during the operative procedure itself [28]. AI models calibrated for intraoperative deployment ingest high-frequency hemodynamic parameters (mean arterial pressure, cardiac output, mixed venous saturation, near-infrared spectroscopy cerebral oxygenation) alongside perfusion metrics and surgical event markers [45,56].

Intraoperative AI-assisted decision support targets two primary domains. First, perfusion optimization, including real-time feedback on oxygen delivery, hemodilution management, and temperature-flow index relationships to minimize bypass-associated end-organ injury. Proof-of-concept reinforcement learning frameworks for perfusion flow optimization have demonstrated feasibility in experimental settings [56]. Second, hemodynamic optimization, including dynamic threshold-based alerts for clinically significant mean arterial pressure and cardiac output deviations, with AI-assisted pharmacologic recommendation for vasopressor and inotropic management aligned with individualized hemodynamic targets [26].

### 6.3. Postoperative Phase: Risk Surveillance and Early Complication Detection

The postoperative ICU represents the highest-information-density environment of the cardiac surgical encounter and the most actionable window for preventive intervention [26,32]. PCInt’s postoperative surveillance layer integrates continuous hemodynamic monitoring streams, laboratory trajectory analysis, ventilator parameter data, fluid balance accounting, and nursing assessment documentation into a unified risk dashboard that provides real-time, patient-specific probability estimates for defined complication domains [27,39].

LSTM-based sequential monitoring models, trained on high-frequency ICU data from large institutional cohorts, would be particularly suited for this phase, capturing the temporal dynamics of physiologic deterioration with reported performance exceeding threshold-based early warning systems in development cohorts [21]. Risk trajectory alerts would be generated when probability estimates cross predefined action thresholds, automatically populating clinical decision support modules with phase-appropriate intervention protocols. Integration with electronic prescribing systems would enable execution of triggered protocols with minimal manual workflow interruption [22,29].

### 6.4. Institutional Integration: Learning Health Systems in Cardiac Surgery

Full realization of PCInt requires its embedding within the institutional quality architecture of the cardiac surgical program. This integration operates across three levels. At the clinical operations level, PCInt outputs are integrated into daily multidisciplinary ICU rounds through structured risk summary dashboards. At the quality program level, PCInt data streams are linked to existing STS database submission workflows, enabling automated risk-adjusted benchmarking and early identification of institutional outcome signals [13,31]. At the organizational learning level, PCInt performance metrics are reviewed at structured quality leadership intervals, driving iterative refinement of both the analytical and clinical components [33,34].

Consistent with the learning health system principles introduced above, every PCInt-monitored surgical case would contribute to model retraining datasets, outcome attribution analyses, and protocol effectiveness evaluations. Over time, the institutional PCInt system would become progressively more calibrated to the specific patient population, surgical complexity mix, and care delivery patterns of the institution, a potential advantage over externally developed, statically deployed AI tools [34,53].

## 7. Potential Impact on Clinical Outcomes, Healthcare Systems, and Value-Based Care

### 7.1. Clinical Outcomes

The primary clinical rationale for PCInt rests on a well-established premise: early, proactive intervention in physiologic deterioration produces better outcomes than late, reactive intervention after complication establishment. This principle is supported across multiple clinical domains relevant to cardiac surgery. In AKI, early fluid and hemodynamic optimization in patients with rising creatinine trajectories reduces the rate of renal replacement therapy requirement [27,39]. In prolonged mechanical ventilation, early spontaneous breathing trial protocols reduce ventilator days and ventilator-associated pneumonia [40]. In POAF, prophylactic amiodarone and early rate-control protocols initiated at first arrhythmia detection reduce cardioversion requirements and anticoagulation-related bleeding [41,42].

To illustrate the potential scale of benefit if such intervention effects were achieved, simulation modeling applied to AI-enhanced AKI prediction in cardiac surgery has estimated that a hypothetical 20% reduction in severe AKI incidence would translate to on the order of several thousand prevented renal replacement therapy episodes annually in the United States [27,37]. Analogous modeling for prolonged ventilation reduction suggests recoverable ICU-days at the population level [32,40]. These figures are illustrative projections contingent on an assumed effect size, not observed results; no such effect has been demonstrated for PCInt or for any integrated AI-linked preventive pathway. Prospective randomized or stepped-wedge evidence for PCInt as an integrated framework is an essential future research priority, and the existing evidence base provides a translational rationale that motivates such investigation rather than evidence of benefit in itself [4,18].

### 7.2. Healthcare Economics and Value-Based Care

The economic implications of PCInt are directly aligned with the value-based care architecture increasingly governing cardiac surgical reimbursement [10]. Under bundled payment models, the financial incentive for complication prevention is explicit: each avoided major adverse event directly improves the episode payment margin. ICU LOS reduction, early extubation, and prevention of renal replacement therapy represent the highest-value outcome improvements available to cardiac surgical programs under value-based contracting [11,57].

In principle, a framework that reduced the incidence of high-cost complications such as AKI requiring renal replacement therapy, prolonged ventilation, and unplanned ICU readmission would lower per-episode costs under bundled payment, and the magnitude of any such savings would scale with surgical volume. We do not attempt to quantify these savings here; absent prospective implementation data, specific cost-savings or return-on-investment estimates would be speculative, and the relevant figures would depend heavily on institutional case mix, baseline complication rates, and implementation expenditure [11,12,58].

### 7.3. Institutional Strategy: Cardiac Surgery as a Model for Predictive Medicine

Cardiac surgery’s combination of high procedural complexity, rich perioperative data infrastructure, high-stakes outcomes, and established quality measurement architecture makes it an ideal domain for the first institutional implementations of PCInt and, more broadly, for the proof-of-concept demonstration of AI-driven preventive medicine at the institutional level [25,31]. The capabilities developed for cardiac surgical PCInt (real-time physiologic monitoring integration, ML-based risk prediction, structured decision support protocols, and learning health system infrastructure) are directly transferable to other high-acuity surgical and medical domains such as aortic surgery, heart transplantation, left ventricular assist device management, and critical care medicine [33,53].

## 8. Challenges, Limitations, and Future Directions

### 8.1. Data Integration and Infrastructure

The most immediate technical challenge facing PCInt implementation is data integration. Cardiac surgical programs operate within complex, heterogeneous data ecosystems (e.g., multiple EHR platforms, proprietary monitoring hardware, standalone perfusion documentation systems, imaging archives, and laboratory information systems), frequently with limited interoperability and inconsistent data quality [29,30]. The technical requirements (HL7 FHIR interfaces, middleware integration engines, structured data quality pipelines, and real-time analytics platforms) represent a substantial institutional investment. Although these capabilities increasingly fall within the reach of academic medical center informatics departments and commercial health AI vendors, the realistic barriers should not be understated: dedicated data-engineering and data-science staffing, clinical informatics governance, ongoing model maintenance, and integration with proprietary vendor systems each carry recurring cost and personnel requirements that many programs, particularly smaller or resource-limited centers, may be unable to sustain. The feasibility, total cost of ownership, and staffing model for institutional-scale deployment are themselves open questions that would require formal implementation-science evaluation rather than assumption [59,60,61,62,63,64,65].

### 8.2. Model Validation and Generalizability

The AI literature in cardiac surgery is characterized by a persistent gap between model development and clinical deployment. The majority of published models are developed and validated within single institutional datasets, raising substantial concerns about generalizability to different patient populations, operative volumes, bypass practices, and care protocols [4,48]. External validation in multi-institutional prospective datasets is an essential prerequisite for clinical deployment, yet it remains the exception in the published literature [18]. PCInt implementation would need to prioritize prospective model validation, with performance monitoring protocols that detect calibration drift (the degradation of model accuracy over time as clinical practices, patient populations, and institutional contexts evolve) and trigger recalibration or retraining procedures [66,67,68,69]. Future development and reporting of the component models should be aligned with emerging consensus standards for clinical AI, including TRIPOD + AI [70] for prediction-model development and validation, PROBAST/PROBAST + AI [71,72] for risk-of-bias appraisal, DECIDE-AI [73] for early-stage clinical evaluation of decision-support systems, and SPIRIT-AI [74] and CONSORT-AI [75] for the protocols and reports of any prospective trials. Adherence to these standards, together with explicit attention to temporal validation, external validation, distributional shift, and post-deployment performance monitoring, would provide the transparency required to judge whether reported performance is likely to persist in routine practice.

A further caution concerns the interpretation of the performance metrics reported throughout this review and summarized in Table 2. The area under the receiver operating characteristic curve, sensitivity, and specificity values cited from the primary literature should be read as the figures reported by the original investigators rather than as established or directly comparable facts. High apparent accuracy in clinical machine learning frequently reflects methodological artifacts rather than genuine predictive capability, including data leakage between training and test partitions, overfitting to institution-specific signal, inappropriate or absent cross-validation, and inadequate handling of class imbalance, which is pervasive in cardiac surgical datasets where major complications are relatively rare events. Many publications do not report learning curves, convergence behavior, or validation-loss trajectories, leaving readers unable to exclude these problems. Reported metrics that are not accompanied by such transparency, by calibration assessment, and by clearly independent test data should therefore be interpreted conservatively, and aggregate performance claims across heterogeneous studies warrant particular skepticism.

The concept of external validation itself warrants deeper scrutiny than it is usually given. The degree to which a validation cohort is truly external matters considerably, and claims of external validation in the literature should be evaluated critically for genuine independence. A validation dataset drawn from a demographically similar patient population, using the same monitoring equipment and manufacturers, with annotators trained in the same conventions and technicians following comparable protocols, provides far weaker evidence of generalizability than the term external validation implies. Readers should therefore not take external validation claims at face value; the relevant question is not whether a second dataset was used, but how far that dataset departs from the development setting along these dimensions.

### 8.3. Explainable AI and Clinician Trust

A critical barrier to clinician adoption of AI-based decision support is the opacity of many high-performance ML models, the “black box” problem. Gradient boosting and neural network architectures that produce the highest predictive accuracy are frequently the least interpretable to clinical end-users [49,51]. The field of explainable AI (XAI), encompassing techniques such as SHAP (SHapley Additive exPlanations) and LIME (Local Interpretable Model-agnostic Explanations), has produced tools that generate patient-specific feature importance explanations for individual predictions [52]. PCInt implementation should prioritize XAI integration as a core design requirement, not a post hoc addition, recognizing that clinician trust and protocol adherence are prerequisites for outcome improvement that accurate prediction alone cannot achieve [51].

### 8.4. Regulatory and Governance Considerations

AI-based clinical decision support tools in the United States are subject to regulatory oversight by the Food and Drug Administration (FDA) under the Software as a Medical Device (SaMD) framework. The regulatory landscape is actively evolving, with the FDA’s predetermined change control plan (PCCP) framework providing a pathway for adaptive AI/ML-based SaMD that updates based on real-world performance data [54]. PCInt implementations must engage regulatory counsel early in the design process, develop prospective performance monitoring protocols aligned with FDA expectations, and maintain documentation architectures appropriate for post-market surveillance requirements [66].

### 8.5. Ethical Considerations, Equity, and Algorithmic Fairness

AI systems trained on historically collected clinical data inherit the structural inequities embedded in that data. If cardiac surgical cohorts used for model development systematically underrepresent women, racial minorities, or patients from underserved healthcare systems, the resulting models may perform differentially across these populations [49,54]. Landmark work has documented racial bias in healthcare algorithms used to manage high-risk populations, demonstrating that algorithmic outputs calibrated to surrogate endpoints can systematically underestimate the burden of illness in minority patients [49]. PCInt implementation must incorporate prospective algorithmic fairness evaluation, including stratified performance assessment across demographic and socioeconomic subgroups, as a standing component of the institutional learning system [48,67].

### 8.6. Digital Twins and Personalized Surgical Simulation

On the horizon of preventive cardiovascular intelligence lies the concept of the surgical digital twin: a continuously updated, patient-specific computational model that integrates anatomical, physiological, pharmacological, and procedural data to simulate individualized surgical outcomes and optimization strategies prior to operative intervention [68,69]. Digital twin technology, currently most advanced in aortic surgery simulation and structural heart disease intervention planning, represents the logical extension of PCInt’s risk trajectory analytic framework into the domain of individualized preoperative strategy [68]. Early proof-of-concept studies in cardiac surgical simulation suggest that digital twin models can identify patient-specific perfusion optimization targets and predict individualized responses to pharmacologic interventions with clinically meaningful accuracy [33,69]. The integration of digital twins into PCInt’s preoperative risk stabilization protocols represents a transformative future direction, one that would enable truly personalized preventive cardiovascular intelligence at the level of the individual surgical encounter.

### 8.7. A Staged Pathway from Concept to Clinical Deployment

Because PCInt is a proposal rather than a validated system, its credibility depends less on the architecture described above than on a realistic account of how it could be evaluated, and we therefore outline a staged pathway from concept to clinical deployment. The first stage would be retrospective model development with internal and temporal (out-of-time) validation on institutional cohorts. The second would be external validation across multiple institutions to test geographic and case-mix generalizability. The third would be silent prospective (shadow) deployment, in which models run on live data and generate predictions that are recorded in the background, allowing prospective calibration assessment without patient exposure. The fourth would be human-factors and alert-burden testing of the decision-support interface, since alarm fatigue is among the most consequential failure modes of intensive care surveillance technology. The fifth would be prospective impact evaluation through pragmatic randomized trials or stepped-wedge cluster implementation studies, which are the designs best suited to determining whether linking prediction to protocolized action actually changes outcomes. Throughout, model performance, fairness, and data drift would be monitored under a predetermined change-control plan, with explicit attention to automation bias and unintended consequences.

This pathway also clarifies why many promising models never reach clinical adoption. Most stop at the first or second stage: they are developed and perhaps internally validated, but are seldom linked to an intervention and are almost never tested prospectively for clinical impact, so that strong retrospective discrimination coexists with an absence of evidence that patient outcomes improve. Recognizing this translational gap is, in our view, more useful than any single performance figure. The principal evidence gaps, the study designs suited to closing them, and the priority research questions they raise are summarized in Table 3.

### 8.8. Limitations

Several limitations of this work should be acknowledged. First, this is a narrative rather than a systematic review; although our search strategy and selection criteria are stated in the Methods, study selection involved author judgment, no formal risk-of-bias appraisal or quantitative synthesis was performed, and relevant publications may have been omitted. Readers should not infer that an unreferenced study was judged to be of lower quality. Second, and most importantly, the Preventive Cardiovascular Intelligence framework presented in Section 5 and Section 6 is a conceptual proposal. It has not been implemented, prospectively tested, or empirically validated as an integrated system, and the projected clinical and economic benefits described are hypotheses derived from the performance of individual component technologies rather than evidence for the framework as a whole. Third, the performance figures synthesized from the primary literature are subject to the methodological caveats detailed in Section 8.2 and may overstate real-world performance. Fourth, the framework presupposes data integration, monitoring, and informatics infrastructure that is unevenly available across institutions; its feasibility in resource-limited settings and its behavior in the presence of substantial missing data remain untested and may be considerably more constrained than the idealized description suggests. Fifth, the equity and fairness risks discussed in Section 8.5 are inherent to any system trained on historical clinical data and would require active, ongoing mitigation rather than one-time evaluation. These limitations define the principal directions for future empirical work.

## 9. Conclusions

Cardiac surgery remains among the highest-risk procedural domains in modern medicine. Despite decades of technical advancement, pharmacological optimization, and systematic quality improvement, major adverse events continue to affect a substantial proportion of operative patients, generating enormous clinical, economic, and humanitarian burden [1,12]. The prevailing paradigm for managing this burden is fundamentally reactive: built on static preoperative risk scores, fragmented perioperative data ecosystems, and retrospective quality review mechanisms that identify complications after their occurrence rather than preventing them before their onset [5,13].

Artificial intelligence offers a genuinely transformative opportunity in this context. AI functions not merely as a supplement to existing static tools, but as a potential analytical substrate for a new operational philosophy. The evidence base for AI in cardiac surgical outcome prediction is substantial and accelerating: ML models reported to improve discrimination relative to conventional risk scores across a range of major postoperative complications; deep learning architectures enabling real-time physiological surveillance; and emerging reinforcement learning frameworks for dynamic intraoperative optimization. These gains are predominantly in predictive discrimination and remain to be translated into demonstrated clinical benefit [4,6,7,56].

What is missing from the existing paradigm is arguably not predictive accuracy but institutional integration. Preventive Cardiovascular Intelligence is proposed to provide such an architecture. By framing cardiac surgical perioperative care as a continuously monitored, dynamically predicted, and proactively managed process, embedded within an institutional learning system that improves with each clinical encounter, PCInt is intended to reframe the role of AI in cardiac surgery, shifting the emphasis from documenting risk toward acting to reduce it [33,34,53].

The implementation of PCInt at the institutional level would require sustained investment in data infrastructure, interdisciplinary collaboration, and a cultural commitment to the primacy of prevention over reaction. These are not trivial requirements, and the framework remains, at present, a hypothesis. Realizing it will depend on prospective, multi-institutional validation of its component models, rigorous evaluation of calibration and fairness across diverse populations, and pragmatic implementation studies that measure whether linking prediction to protocolized action actually changes patient outcomes. If these conditions are met, the alignment of preventive outcome improvement with the incentives of value-based surgical care offers a coherent rationale for the investment; until they are, the claims advanced here should be regarded as a research agenda rather than a demonstrated result.

## Figures and Tables

**Table 1:** Comparison of Traditional Risk Models vs. AI-Driven Preventive Cardiovascular Intelligence (PCInt) Framework. M&M: Morbidity and mortality.

Domain	Traditional Risk Models (STS/EuroSCORE)	AI-Driven PCInt Framework
Timing	Preoperative only	Continuous perioperative
Data inputs	Clinical variables at a single time point	Multimodal, real-time data streams
Prediction target	In-hospital mortality, major morbidity	Individualized, dynamic complication risk
Actionability	Risk categorization (low/medium/high)	Triggered preventive intervention protocols
Feedback loop	Annual registry benchmarking	Real-time institutional learning system
Patient granularity	Population-level risk estimates	Patient-specific risk trajectories
Modality integration	Limited (demographics, labs, procedures)	EHR, imaging, wearables, perfusion data
Response time	Retrospective (M&M conference, audit)	Prospective and proactive
Clinical culture	Reactive complication management	Preventive cardiovascular intelligence

**Table 2:** Current AI Applications in Cardiac Surgery.

Application	Representative Methodology	Prediction Window	Reported Performance (Metric Type)	Development and Validation Status	Prospectively Implemented and Linked to Intervention?
AKI prediction	Gradient boosting (XGBoost, LightGBM)	Postoperative	AUROC 0.80–0.90 (discrimination) [37,38,39]	Predominantly single-center development with internal validation; external validation uncommon	No
Mortality prediction	Neural networks, penalized regression	Pre-/postoperative	Improved discrimination vs. STS PROM in reported series [16,17,18]	Single- and multi-center development; external validation limited	No
Prolonged mechanical ventilation	Random forest, gradient boosting	Postoperative ICU	Sensitivity >80% at selected thresholds [40]	Single-center development; internal validation	No
Atrial fibrillation (POAF)	Deep learning (ECG-based)	Perioperative	Early detection up to ~24 h before onset (discrimination) [19,20,43]	Single-center development; internal validation	No
ICU deterioration	LSTM, recurrent neural networks	Real-time monitoring	Earlier flagging than MEWS/NEWS in development cohorts [26]	Single-center development; internal validation	Rare
Transfusion requirement	Regression + ML ensembles	Intraoperative	Discrimination for transfusion need; no prospective outcome data [44,45,46,47,48,49,50]	Single-center development	No
Hospital readmission	Gradient boosting, random forest	Discharge planning	AUROC 0.75–0.85 (discrimination) [29,36]	Single- and multi-center development; external validation limited	No
Stroke/neurological events	Bayesian networks, CNN	Perioperative	Emerging, limited evidence [19]	Early-stage development	No
Perfusion optimization	Reinforcement learning	Intraoperative	Proof-of-concept/feasibility (experimental) [51,52,53,54,55,56]	Pre-clinical/experimental	No
NLP for QI/outcome coding	NLP (BERT-based)	Registry/EHR mining	Automated outcome coding (feasibility) [35]	Development; internal validation	No

Entries summarize the general state of each application area rather than individual studies; sample sizes, cohorts, and exact validation procedures are reported in the cited primary sources. Reported performance reflects predictive discrimination (or, where stated, sensitivity) in development or internal-validation cohorts and should not be interpreted as evidence of improved clinical outcomes. The final column indicates whether, to our knowledge, the application has been deployed prospectively and linked to a clinical intervention in cardiac surgery; in most areas this remains rare or absent. AUROC, Area Under the Receiver Operating Characteristic Curve; CNN, Convolutional Neural Network; ECG, Electrocardiogram; EHR, Electronic Health Record; ICU, Intensive Care Unit; LSTM, Long Short-Term Memory; MEWS, Modified Early Warning Score; ML, Machine Learning; NEWS, National Early Warning Score; NLP, Natural Language Processing; POAF, Postoperative Atrial Fibrillation; PROM, Predicted Risk of Mortality; QI, Quality Improvement; STS, Society of Thoracic Surgeons; AKI, acute kidney injury.

**Table 3:** Priority evidence gaps, recommended study designs, and research questions for AI-enabled preventive perioperative care in cardiac surgery. AI, artificial intelligence; ICU, intensive care unit; EHR, electronic health record.

Evidence Gap	Current Status	Recommended Study Design	Priority Research Question
External and temporal validation of component models	Most models are single-center and internally validated; genuine external validation is rare	Multi-institutional external validation and temporal (out-of-time) validation, reported per TRIPOD + AI and appraised with PROBAST-AI	Do reported discrimination and calibration estimates hold across institutions, equipment, and time?
Calibration and clinical usefulness	Calibration is under-reported and often assessed only with the Hosmer-Lemeshow statistic	Calibration-in-the-large, calibration slope, calibration plots, Brier score, and expected calibration error, with decision-curve analysis at defined thresholds	Are predicted probabilities reliable and net-beneficial enough to guide action at clinically relevant thresholds?
The prediction-to-action link	Most models conclude at a discrimination statistic and are not linked to any intervention	Silent prospective (shadow) deployment followed by alert-linked protocol pilots	Does linking prediction to a structured intervention change clinician behavior and intermediate outcomes?
Prospective clinical impact	Prospective or randomized evidence of outcome benefit is almost entirely absent	Pragmatic randomized trials or stepped-wedge cluster implementation studies	Does an AI-linked preventive pathway reduce complications, ICU days, or cost versus usual care?
Alert burden and human factors	Alarm fatigue is a known failure mode but is rarely quantified for surgical AI	Human-factors and usability testing, alert-burden and workflow studies (DECIDE-AI for early clinical evaluation)	What alert design and tiering preserve sensitivity while keeping clinician burden acceptable?
Composite prioritization (Risk Burden Index)	A proposed construct; weighting, correlation handling, and fairness are unresolved	Retrospective simulation against existing datasets, sensitivity analysis of weights, and subgroup calibration	Can a composite index prioritize concurrent risk without double-counting or introducing inequity?
Equity and algorithmic fairness	Models may perform differentially across demographic and socioeconomic subgroups	Pre-specified subgroup performance and calibration auditing, bias mitigation, and ongoing monitoring	Does the system perform equitably, and are disparities detected and corrected over time?
Drift and post-deployment monitoring	Performance degrades as practice patterns and populations evolve	A predetermined change-control plan with continuous calibration-drift surveillance and recalibration triggers	How is sustained performance ensured and governed after deployment?
Infrastructure, interoperability, and cost	EHR, monitoring, and perfusion systems are heterogeneous and integration is costly	Implementation-science evaluation of interoperability, staffing requirements, and total cost of ownership	Is institutional-scale integration technically and economically feasible, and at what cost?

## Data Availability

No new data were created for the present manuscript.

## Abbreviations

The following abbreviations are used in this manuscript:

AI	Artificial Intelligence
AHRQ	Agency for Healthcare Research and Quality
AKI	Acute Kidney Injury
AIMS	Anesthesia Information Management System
ARR	Annual Recurring Revenue
AUROC	Area Under the Receiver Operating Characteristic Curve
CABG	Coronary Artery Bypass Grafting
CNN	Convolutional Neural Network
CMS	Centers for Medicare and Medicaid Services
EHR	Electronic Health Record
EU MDR	European Union Medical Device Regulation
EuroSCORE	European System for Cardiac Operative Risk Evaluation
FDA	Food and Drug Administration
ICU	Intensive Care Unit
IP	Intellectual Property
LSTM	Long Short-Term Memory
LVAD	Left Ventricular Assist Device
MEWS	Modified Early Warning Score
ML	Machine Learning
MVP	Minimum Viable Product
NIH	National Institutes of Health
NLP	Natural Language Processing
PCCP	Predetermined Change Control Plan
PCInt	Preventive Cardiovascular Intelligence
PCORI	Patient-Centered Outcomes Research Institute
POAF	Postoperative Atrial Fibrillation
QI	Quality Improvement
RNN	Recurrent Neural Network
RRT	Renal Replacement Therapy
SaaS	Software as a Service
SaMD	Software as a Medical Device
SAVR	Surgical Aortic Valve Replacement
STS	Society of Thoracic Surgeons
STTR	Small Business Technology Transfer
XAI	Explainable Artificial Intelligence
XGBoost	Extreme Gradient Boosting
